# Medical Students’ Expectations Towards an Implementation of a Family Medicine Textbook as a Comprehensive App in Germany

**DOI:** 10.1007/s10916-014-0125-y

**Published:** 2014-08-30

**Authors:** Maximilian Sandholzer, Imre Rurik, Tobias Deutsch, Thomas Frese

**Affiliations:** 1 Institute for Medical Informatics, Statistics and Epidemiology, University of Leipzig, Härtelstraße 16-18, 04107 Leipzig, Germany; 2 Department of Family and Occupational Medicine, Faculty of Public Health, University of Debrecen, Debrecen, Hungary; 3 Department of Primary Care, Leipzig Medical School, University of Leipzig, Leipzig, Germany

**Keywords:** Smartphone, Elearning, Ehealth, General practice, App specifications

## Abstract

Undergraduate and postgraduate medical education in general practice is complex as a wide medical spectrum needs to be covered. Modern guidelines demand students to be able to recall immense amounts of information relating to the diagnosis and management of clinical problems. With the intent of making a medical textbook digitally available on student mobile devices, preferences of students and potential of the idea was aimed to be researched. A total estimation among fourth year medical students at the Leipzig Medical School was conducted in June 2013. Students were asked to answer a semi-structured self-designed questionnaire regarding their detailed smartphone and app usage as well as their attitude and expectations towards education and practice supporting apps. The response rate was 93.2 % (*n* = 290/311). The majority (69.3 %) were female students. The mean age was 24.5 years. Of the respondents, 64.2 % owned a smartphone and 22.5 % a tablet computer. A total of 32.4 % were already using medical apps for the smartphone - mostly drug reference or disease diagnosis and management apps. Regarding their wishes, 68.7 % would like or very like to see an app on general practice. The respective means of the most important desired features on a Likert scale reaching from 1 (not important) to 5 (very important) were 4.3 for drug reference information, 4.2 for guidelines for differential diagnosis, 3.9. for medical pictures libraries and 3.9 for physical examination videos. The willingness to pay for a profound app averages at 14.35 Euros (SD = 16.21). Concluding, students clearly demand for an app on general practice. Such an app should ideally be smartphone optimized. Aside of what is usually available in traditional textbooks, multimedia features such as videos on examining methods or a medical picture library are very important to students and may help to bridge the gap between text-based knowledge and practical application. Therefore, authors of medical textbooks need to be aware that the development of an app is no trivial technical translation as raised students expectations demand for multimedia and interactive features as well as comprehensive drug information. Further research should focus on developing concepts to bring together developers and university professionals as well as experienced medical specialists to enable the development of apps that satisfy the demands of undergraduate and postgraduate educational needs.

## Background

Various functions of smartphones and tablet computers such as on-the-go internet access, mailing, navigation, mobile entertainment and many more allow access to information resources and open up new possibilities in medicine. Smartphone-based applications in the field of healthcare have received increasing attention in research lately. For instance, 55 of articles on healthcare applications reviewed by others were published after 2003 and 24 of these between January 2010 and April 2011 [10]. Also research is conducted based in projects that supply medical students with smartphones and suitable medical applications. In September 2010 for instance, medical students at Leeds University received smartphones to enable them to access drug prescription as well as medical textbooks at a cost of £380 per student. In another project called “All-Wales Foundation Programme iDoc Project”, £500,000 were invested to supply junior doctors with smartphones and pre-downloaded medical textbooks [1].

Bottom-line, previous studies have intensively researched how smartphones and mobile health apps are and can be employed by students, doctors and patients by conducting both empirical research as well as in-depth literature reviews [1, 3, 6, 7, 11]. A high focus has been on reviewing the available apps as tools for application areas such as evidence-based medicine or clinical decision support [8]. Reviewed apps include apps for literature search, disease diagnosis, drug reference, medical calculators, clinical calculations and various other kinds. Mobile medical apps and smartphones were found to be widely adopted among doctors and patients [5, 6, 10]. In 2012, a study from the United Kingdom reviewed the smartphone and medical related app usage among medical students and junior doctors using an online survey. Smartphone ownership, types of apps used or owned, frequency and length of app usage were surveyed. The authors of this study called for more research on appropriate app development as well as on the perceptions of mobile technology usage in clinical areas [12]. The effects of smartphone usage in the presence of patients have been assessed, as using a smartphone may result in false perceptions regarding the physician, his work and his care for the patient. In this case it has been concluded that doctors should take the time to explain to patients why they are using their smartphones and share their findings with them [9].

A study from 2011 among respondents enrolled in a training program found that the most frequent app type requested were textbook and reference materials [4]. Especially evidence-based-medicine in a general practice setting calls for up-to-date knowledge of research and treatment methods. In this case, digital sources offer valuable advantages in compare to traditional textbook such as updateability or just the mere convenience advantage due to lower weight and volume. Therefore, at the Leipzig Medical School a textbook called “General Practice - a lecture-adjusted and problem-based Presentation“[13] used in lectures should be made digitally available to the students.

In contrast to previous research, our study should not solely focus on smartphone and other mobile technology usage or ownership, but also on the demands of students towards a medical application. We therefore investigated the following questions: What kind of technical devices do students own and which medical apps do they already use? What is the attitude of students towards an app for general practice^1^ and how should it be designed in terms of content contained and functionality? What would be typical situations to use the app? By answering these questions, our study aims to support the development of a prototype app that specifically supports medicine students in their studies and bridges the gap to the practice in clinical areas. In order to precisely capture the students’ preferences to assure an appropriate app development, a survey was designed.

## Material and Methods

### Questionnaire and Content

In June 2013 we distributed a total of 311 questionnaires (Appendix) by person to medical students of the Leipzig Medical School. The questionnaire was designed by an interdisciplinary team (an economist, a psychologist, two general practitioners) and questions were taken from previous literature [12] and the researchers’ experience. A backward translation of the resulting German items was not performed, because the items were generally understandable and our study was not set out to investigate nation to nation differences in a direct comparison. The study was piloted within a round of eight 4th year medical students. This resulted in minor revisions regarding the content and structure of the questionnaire.

The survey questions recorded data on the following areas: general information (gender, age, etc.), devices owned, medical apps owned (open-entry box), need for a general practice app, desired functionality and content of such an app, situations for which such an app should be suited, willingness to pay and desired update frequency. Additionally, an open text entry box allowed students to submit other comments. The medical apps named in the open-entry box for apps owned and used by students were grouped into five categories: Drug reference apps, medical calculators, disease diagnosis management apps, procedure case documentation, and basic medical science knowledge. These categories are much aligned with Payne et al. [12] for better comparability, except for us adding the category “basic medical science knowledge”. Attributing apps to groups has been done by reviewing app descriptions by two experienced raters.

### Sampling

The questionnaire was distributed after a mandatory 45 min multiple-choice examination of general practice to all fourth year medical students (8th semester) at the Leipzig Medical School (In Germany there is a 6 year medical curriculum. It consists of two pre-clinical years and four clinical education years. The practical year is the final year of undergraduate education. It is a clinical year and divided in three sections: Surgery, internal medicine, one discipline eligible for selection by each student). We choose this student sample, as they are participants of a compulsory course in general practice and thereby are aware of the content of the textbook that is supposed to be implemented as an app. All of these students were briefed beforehand that the questionnaires purpose is to assess what to consider when implementing a digitally available version of the lecture’s textbook. The participation was voluntary. Regarding the regulations of the ethical board of the Leipzig Medical School, no ethical approval was necessary.

### Statistical Analysis

The anonymous data was analyzed with IBM SPSS Statistics 18 for Windows (SPSS Inc., 2009). Frequencies were presented as %_valid_ (n_absolute_/n_valid_), as the number of valid values differed from item to item. Continuous variables were presented as mean ± standard deviation (SD), supplemented by median and interquartile range (IQR) if necessary. Frequencies were compared using the Chi-square test. Differences were stated as statistically significant for *p* < 0.05.

## Results

### Sample Characteristics

In total 290 out of the 311 students surveyed at the Leipzig medical school participated, which is equal to a response rate of 93.2 %. Out of the 290 returned questionnaires 69.3 % (*n* = 201/290) were answered by female students. Mean age of participants was 24.5 years ranging from the age of 21 to the age of 43 with a standard deviation of 2.9 years (*n* = 284).

### Device Ownership and Medical App Usage

**Fig. 1:**
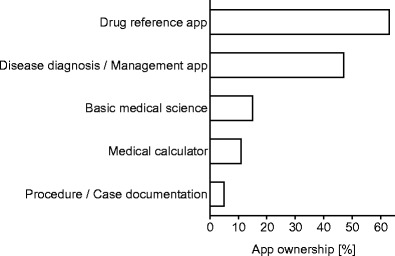
The result of grouping the used apps into the five different categories and the distribution of its frequencies among students who regularly use apps

Of the medical students surveyed, 64.2 % (*n* = 183/285) owned a smartphone, 88.8 % (*n* = 253/285) owned a laptop, 22.5 % (*n* = 64/285) owned a tablet computer and only 21.4 % (*n* = 61/285) owned an ordinary desktop computer. Additionally, 82.8 % (*n* = 53/64) of the tablet computer owners used a smartphone. We found no differences towards the ownership of smartphone or tablet computer between male and female students (71.3 % (*n* = 62/87) vs. 66.7 % (*n* = 132/198); *p* = 0.443). Students were also asked to answer whether they have a private internet access possibility, which 81.4 % (*n* = 232/285) affirmed. When asked about their medical app usage, 32.4 % (*n* = 92/284) of the students answered yes, they use medical apps. Male students reported more frequently to use apps than female students (43.2 % (*n* = 38/88) vs. 27.6 % (*n* = 54/196); *p* = 0.009). Furthermore, they were asked to specifically name the apps they were using. The result of grouping the used apps the different categories is displayed in Fig. 1.

### Attitude Towards an App for General Practice and its Functionality

**Fig. 2:**
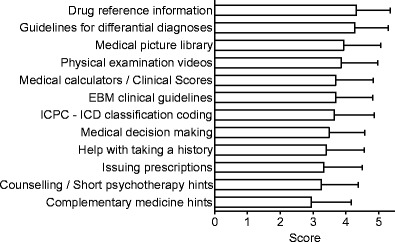
The average score (mean ± SD) towards the functionality of a general practice app based a Likert scale reaching from 1 (unimportant) to 5 (very important; n ranges from 247 to 252 due to missing answers)

In total 68.7 % (*n* = 191/278) of the students declared that they would like or very like to have an app on general practice. This corresponds to a mean of 3.8 ± 1.4 (*n* = 278) on a Likert scale reaching from 1 (strongly disagree) to 5 (strongly agree). Students currently owning a smartphone or tablet computer would like or very like to have an app on general practice more frequently than others (82.4 (*n* = 159/193) vs. 39.0 % (*n* = 32/82); *p* < 0.001). 53.8 % (*n* = 134/249) of the surveyed stated that the app should contain content from all commonly used medical textbooks available on the market (choosing 4 or 5 on a 5 point Likert scale from 1 = “strongly disagree” to 5 = “strongly agree”). Also, 46.6 % (*n* = 118/253) stated, that the app should additionally be optimized for tablet computers (choosing 4 or 5 on a 5 point Likert scale from 1 = “strongly disagree” to 5 = “strongly agree”). Regarding the frequency of content updates, 61.8 % (*n* = 141/228) would want it to be at least once a year while average requested update interval is 1.4 (±0.9) years. When updated every half year, 96.9 % (*n* = 221/228) of the surveyed students are satisfied. The answers regarding the question of the desired functionality of such an app are summarized in Fig. 2.

### Situations to Use App

The practical year with 81.1 % (*n* = 202/249) is the most frequently named situation in which students want to employ the app, followed by the internship and the mandatory clerkship with 69.9 % (*n* = 174/249) and 69.1 % (*n* = 172/249) respectively. Also, 55.4 % (*n* = 138/249) of the surveyed students would like to use the app for final exam preparation. The preparation of lectures was mentioned by 47.8 % (*n* = 119/249) of the respondents as a situation to use the app. Furthermore, students had the possibility to name other situations, which 14.2 % (*n* = 35/247) of them did. The free text answers can mostly be summarized as learning on the go and checking for information in everyday life, if, for instance, fellow students and friends have questions regarding a medical topic. On a Likert scale reaching from “1 strongly disagree” to “5 strongly agree”, 67.1 % (*n* = 169/252) of the students selected a 4 or higher regarding the question whether the app should support their vocational training. Also, on the identical scale, 42.2 % (*n* = 106/251) selected 4 or higher stating that they want the app to aid them when opening an own doctors office.

At last students were asked to name a price they are willing to pay for a profound app on general practice. Named prices average at 14.35 Euros with a median of 10.00 Euros (SD = 16.21, IQR = 15.01 Euros) and ranged as high as 100 Euros (*n* = 204). For the interviewed student group, a price of 10 Euros reflects 53.4 % (*n* = 109/204) and a price of 5 Euros reflects 77.0 % (*n* = 157/204) of the student’s willingness to pay. Also, a total of 19 students equaling 9.3 % (*n* = 19/204) called for the app to be free of any charge. In a free comment box located at the end of the survey, some students wished for the app to be available on at least all the common platforms such as Android and iOS.

## Discussion

### Main Findings

Our study demonstrates that medical students demand not only an app for educational purposes such as exam preparation, but for an app for undergraduate and post-graduate medical education that accompanies them in daily practice. The recent work is the first of its kind providing a sufficient sample size and response rate.

### Discussion with Findings from the Literature

As it was reported from another German investigation with different design and only a small number of responders, medical students seem to use smartphones regularly for professional purposes [14]. We found smartphone ownership among German medical students to be lower than reported by other studies, who have discovered ownership rates among medical students as high as 79 % in the UK and 77 % in Australia [7, 12]. Our data suggest that tablet ownership may increase from its current state: Only 22.1 % (*n* = 63/290) of the students owned a tablet computer but 46.6 % (*n* = 118/253) wished for an app to be additionally optimized for tablet computers. Koehler and Yao reported that 3 % of students indicated that they do not consider obtaining a medical app or a smartphone so that they can use medical apps. All others that did not own a smartphone yet said they were planning to obtain one to gain access to medical applications [7]. Our discovered medical app usage of 32.4 % is by far lower than the 79.8 % in the UK and the 63.6 % in Australia [7, 12]. Beside other reasons it can be assumed that the chosen sampling method (total estimation vs. online survey) and the resulting different response rates (93.2 % vs. less than 25 %) may contribute to the estimated difference.

We found that the majority of the students would welcome an app on general practice specifically related to their studies. This is also supported by Payne and colleagues [12], who mentioned a clear trend of students who would like to see an app specifically related to their university. Yet, they are demanding towards the apps content and service provided to users. Our results clearly indicate that students do not just want their textbook to be digitally available as an application with some additional features, but call for interactive and media content such as access to medical picture or examination video libraries and guidelines for differential diagnosis. This is in line with a recent study that has reviewed smartphone applications for mobile clinical decision support systems. The authors concluded that reviewed apps provided a user experience similar to the one of reading a book and developers should take advantage of the possibilities of smartphones to develop more interactive applications [8].

Regarding the kind of apps used, we found drug reference as the most important content feature of such an app and disease diagnosis and management apps as very popular among students. This is in comply with what others reported [10, 12]. Drug prescriptions demand for a high secureness of information given at a high level of detail. Yet, already plenty of high quality drug reference apps are available. Therefore, it could be inferred that students would prefer not to have different apps for different situations, topics and use-cases but rather one comprehensive app. This is also supported by the situations named in which they want to use the app. Students want the app to be both suited for practical work and educational purposes, however other research reported students’ concerns of using an app during consultations, home visits or spare moments [14]. As they named the practical year, the internship and the mandatory clerkship as the most important times in which they would like to employ the app, it can be inferred that it needs to be specifically tailored to these periods of medical education and the resulting tasks. This is supported by the reported results of Payne and colleagues [12] who showed that the demands of smartphone-based support differed between medical students and junior doctors. Thereby it is interesting that half of the students demanded for an app that supports them in founding an own office.

When compared to the situations named above, it is noteworthy that only a relatively small fraction, 55.4 % of the students, said they want to use the app for final exam preparation. This can be due to smartphone screens being relatively small and while studying at one fix location traditional textbooks are handier as a reference tool while the benefit of a smartphone application comes to light when being on-the-go or between classes, as they are more portable [7]. Considering that we interviewed students who were in the 4th year, thus before their 6th purely clinical year, one could expect that learning needs for the university such as preparation of exams would dominate the attitude towards a smartphone app.

### Strengths and Limitations

According to our knowledge, this study is the first study examining the attitude and wishes of student towards a transformation of a medical textbook into an app for education and use in practice. A clear strength of this study is the high response rate combined with a written questionnaire, which reduces the risk of non-response bias and voluntary response bias as mentioned as a limitation of Payne and colleagues [12]. Thus, the higher likeliness of smartphone or tablet users to answer a survey regarding mobile devices and apps can be neglected. A clear limitation of the study is that some of the recorded figures such as smartphone ownership or medical application usage capture a snapshot of a current point in time of a topic which is evolving rapidly. The current study was limited to one German university. Despite a different study design we found a high accordance to the findings of Payne and colleagues [12]. This indicates that our results may be extrapolated to other settings and thereby strengthens our investigation.

### Implications for Education and Future Research

Our survey clearly shows that students want to use an app - albeit their daily work is mainly attending lectures - that helps them later as a bedside teacher solving clinical problems. This means that an app must provide much more information than necessary for undergraduate education. As a consequence we have to expand the content of the textbook to be adapted to an internet based resource to fit in the expectations of future doctors.

Further research should focus on developing concepts to bring together developers and university professionals as well as experienced doctors to enable the development of apps that assure a high-quality content combined with a user-friendly interface. Also, as we found significant differences in internationally reported app usage, it would be necessary to promote international collaborative research of medical students and doctors to account for the demands regarding an app on general practice at different points in the medical education. This would contribute to our findings on how students perceive an app that accompanies them from education to practice.

Additionally, such an app can have a huge potential in creating trust of future, economically important consumers of health care information and products. For instance most of the marketing of drugs is directed to doctors settled in private practice, which have to be convinced to trust in the information delivered via drug company representatives, medical journals and others. A point worthwhile to investigate is that when students use such an app from study to vocational training, one could expect that they perceive the given information as highly trustworthy.

## Conclusions

Students clearly demand for an app on general practice. With significantly more students owning smartphones rather than tablet computers, such an app should ideally be smartphone optimized. Aside of what is usually available in traditional textbooks, multimedia features such as videos on examining methods or a medical picture library are very important to students. We found that students want to use an app that helps them later as a bedside teacher solving clinical problems.

## Footnotes

1 We use the term „general practice“as a synonym for „family medicine“, and „general internal medicine“as terms vary from country to country. For further information on the German medical education system please refer to Chenot, J. F. (2009). “Undergraduate medical education in Germany.” Ger Med Sci 7: Doc02 [2].
